# Health Literacy, eHealth Literacy, Adherence to Infection Prevention and Control Procedures, Lifestyle Changes, and Suspected COVID-19 Symptoms Among Health Care Workers During Lockdown: Online Survey

**DOI:** 10.2196/22894

**Published:** 2020-11-12

**Authors:** Binh N Do, Tien V Tran, Dung T Phan, Hoang C Nguyen, Thao T P Nguyen, Huu C Nguyen, Tung H Ha, Hung K Dao, Manh V Trinh, Thinh V Do, Hung Q Nguyen, Tam T Vo, Nhan P T Nguyen, Cuong Q Tran, Khanh V Tran, Trang T Duong, Hai X Pham, Lam V Nguyen, Kien T Nguyen, Peter W S Chang, Tuyen Van Duong

**Affiliations:** 1 Department of Infectious Diseases Vietnam Military Medical University Hanoi Vietnam; 2 Division of Military Science Military Hospital 103 Hanoi Vietnam; 3 Director Office Military Hospital 103 Hanoi Vietnam; 4 Faculty of Nursing Hanoi University of Business and Technology Hanoi Vietnam; 5 Nursing Office Viet Duc University Hospital Hanoi Vietnam; 6 Director Office Thai Nguyen National Hospital Thai Nguyen Vietnam; 7 President Office Thai Nguyen University of Medicine and Pharmacy Thai Nguyen Vietnam; 8 Health Management Training Institute Hue University of Medicine and Pharmacy Thua Thien Hue Vietnam; 9 Department of Health Economics Corvinus University of Budapest Budapest Hungary; 10 Department of Thoracic and Cardiovascular Surgery E Hospital Hanoi Vietnam; 11 Director Office E Hospital Hanoi Vietnam; 12 Director Office General Hospital of Agricultural Hanoi Vietnam; 13 Director Office Bac Ninh Obstetrics and Pediatrics Hospital Bac Ninh Vietnam; 14 Director Office Quang Ninh General Hospital Quang Ninh Vietnam; 15 Director Office Bai Chay Hospital Quang Ninh Vietnam; 16 Director Office Quang Ninh Obstetrics and Pediatrics Hospital Quang Ninh Vietnam; 17 Director Office Trieu Phong District Health Center Quang Tri Vietnam; 18 General Planning Department Da Nang Oncology Hospital Da Nang Vietnam; 19 Director Office Thu Duc District Health Center Ho Chi Minh Vietnam; 20 Faculty of Health Mekong University Vinh Long Vietnam; 21 Director Office Hospital District 2 Ho Chi Minh Vietnam; 22 Nursing Office Tan Phu District Hospital Ho Chi Minh Vietnam; 23 Director Office District 9 Health Center Ho Chi Minh Vietnam; 24 President Office Can Tho University of Medicine and Pharmacy Can Tho Vietnam; 25 Aesthetic Plastic Surgery & Skin Care Center Can Tho University of Medicine and Pharmacy Hospital Can Tho Vietnam; 26 Department of Health Education Faculty of Social Sciences, Behavior and Health Education Hanoi University of Public Health Hanoi Vietnam; 27 School of Medicine Chung Shan Medical University Taichung Taiwan; 28 Department of Public Health and Community Medicine Tufts University School Of Medicine Boston, MA United States; 29 School of Nutrition and Health Sciences Taipei Medical University Taipei Taiwan

**Keywords:** COVID-19, health literacy, eHealth literacy, health care workers, personal protective equipment, handwashing, masks, disposing, lifestyle, Vietnam, eHealth, adherence, infection prevention, control

## Abstract

**Background:**

The COVID-19 pandemic has imposed a heavy burden on health care systems and governments. Health literacy (HL) and eHealth literacy (as measured by the eHealth Literacy Scale [eHEALS]) are recognized as strategic public health elements but they have been underestimated during the pandemic. HL, eHEALS score, practices, lifestyles, and the health status of health care workers (HCWs) play crucial roles in containing the COVID-19 pandemic.

**Objective:**

The aim of this study is to evaluate the psychometric properties of the eHEALS and examine associations of HL and eHEALS scores with adherence to infection prevention and control (IPC) procedures, lifestyle changes, and suspected COVID-19 symptoms among HCWs during lockdown.

**Methods:**

We conducted an online survey of 5209 HCWs from 15 hospitals and health centers across Vietnam from April 6 to April 19, 2020. Participants answered questions related to sociodemographics, HL, eHEALS, adherence to IPC procedures, behavior changes in eating, smoking, drinking, and physical activity, and suspected COVID-19 symptoms. Principal component analysis, correlation analysis, and bivariate and multivariate linear and logistic regression models were used to validate the eHEALS and examine associations.

**Results:**

The eHEALS had a satisfactory construct validity with 8 items highly loaded on one component, with factor loadings ranked from 0.78 to 0.92 explaining 76.34% of variance; satisfactory criterion validity as correlated with HL (ρ=0.42); satisfactory convergent validity with high item-scale correlations (ρ=0.80-0.84); and high internal consistency (Cronbach α=.95). HL and eHEALS scores were significantly higher in men (unstandardized coefficient [B]=1.01, 95% CI 0.57-1.45, *P*<.001; B=0.72, 95% CI 0.43-1.00, *P*<.001), those with a better ability to pay for medication (B=1.65, 95% CI 1.25-2.05, *P*<.001; B=0.60, 95% CI 0.34-0.86, *P*<.001), doctors (B=1.29, 95% CI 0.73-1.84, *P*<.001; B 0.56, 95% CI 0.20-0.93, *P*=.003), and those with epidemic containment experience (B=1.96, 95% CI 1.56-2.37, *P*<.001; B=0.64, 95% CI 0.38-0.91, *P*<.001), as compared to their counterparts, respectively. HCWs with higher HL or eHEALS scores had better adherence to IPC procedures (B=0.13, 95% CI 0.10-0.15, *P*<.001; B=0.22, 95% CI 0.19-0.26, *P*<.001), had a higher likelihood of healthy eating (odds ratio [OR] 1.04, 95% CI 1.01-1.06, *P*=.001; OR 1.04, 95% CI 1.02-1.07, *P*=.002), were more physically active (OR 1.03, 95% CI 1.02-1.03, *P*<.001; OR 1.04, 95% CI 1.03-1.05, *P*<.001), and had a lower likelihood of suspected COVID-19 symptoms (OR 0.97, 95% CI 0.96-0.98, *P*<.001; OR 0.96, 95% CI 0.95-0.98, *P*<.001), respectively.

**Conclusions:**

The eHEALS is a valid and reliable survey tool. Gender, ability to pay for medication, profession, and epidemic containment experience were independent predictors of HL and eHEALS scores. HCWs with higher HL or eHEALS scores had better adherence to IPC procedures, healthier lifestyles, and a lower likelihood of suspected COVID-19 symptoms. Efforts to improve HCWs’ HL and eHEALS scores can help to contain the COVID-19 pandemic and minimize its consequences.

## Introduction

COVID-19, the disease caused by SARS-CoV-2, has created unprecedented challenges worldwide [1-6], with significant socioeconomic burdens [7], morbidity, and mortality [8,9]. Multidisciplinary and multidimensional approaches are required to contain the pandemic [10-12]. Social and behavioral changes [13,14] and improving health literacy (HL) [15] and eHealth literacy (as measured by the eHealth Literacy Scale [eHEALS]) [16] are highly recommended to control this global health crisis while effective treatments and vaccines are unavailable.

Lockdown measures were applied in many countries, including Vietnam [17]. Lockdown is a necessary public health approach to contain COVID-19. During the lockdown period, health care workers (HCWs) still needed to work and provide health services to patients, which made them more vulnerable to COVID-19 infection. In addition, the pandemic itself and social distancing measures have had negative impacts on psychological health [18,19] and health-related behavior (eg, unhealthy eating habits and behaviors, less physical activity, and more smoking [20-23] and drinking to cope amid the COVID-19 pandemic [24]).

Online consultations from hospitals and health care centers were found to be a safe and effective way to reduce the negative effects of the pandemic [25]. In addition, social media can provide insights for effective communication among public health authorities [26], researchers [27], and the public [28-30]. However, social media spreads panic and anxiety among the public [31]. Disinformation and misinformation have been raised as highly concerning issues for the public [32-34]. HL plays an important role in evaluating online health information [35]. HL is critical for people who interact with the digital world, with its diverse information and sources [36], especially during the COVID-19 pandemic. HCWs play an important role in supporting the public to combat misinformation and disinformation [28,37]. The HCWs’ consultations cannot be altered by social media networks [37,38]. Continuous training and education have been recognized as effective approaches to improving HCWs’ HL, further improving health care delivery [39,40], communication [39,41,42], shared decision making [43], and patient health outcomes [40]. Furthermore, raising awareness of behavioral pitfalls could support appropriate behavioral changes and containment of the crisis [44]. HL and eHealth literacy are more important than ever due to the COVID-19 pandemic [16,45]. However, these issues have been underestimated during the pandemic [15].

We evaluated the psychometric properties of the eHEALS and examined the predictors of HL and eHEALS scores. We also examined the associations between HL and eHEALS scores with adherence to infection prevention and control (IPC) measures, lifestyle changes, and suspected COVID-19 symptoms among HCWs during the lockdown period in Vietnam.

## Methods

### Study Design and Settings

A cross-sectional study was conducted with HCWs April 6-19, 2020, using online-based questionnaires (Text 1 in Multimedia Appendix 1). The HCWs were recruited from 12 hospitals and 3 health centers across Vietnam, including 8 hospitals in the nation’s north, 1 hospital and 1 health center in the central region, and 3 hospitals and 2 health centers in the country’s south.

### Study Participants and Data Collection

No HCWs (doctors and nurses) in our study had provided any direct care or had contact with patients with COVID-19. A total sample of 5209 HCWs (out of 11,517 possible participants) completed an online survey. The studied and possible participants from public hospitals and health centers are presented in Table 1.

**Table 1:** Participants from the studied hospitals and health centers by geographic location.

Geographic location and hospital/health center			Possible participants	Studied participants
**North**				
	**Ha Noi city**			
		Military Hospital 103	1660	177
		E hospital	1125	335
		General Hospital of Agricultural	555	424
	**Thai Nguyen province**			
		Thai Nguyen National Hospital	1186	988
	**Bac Ninh city**			
		Bac Ninh Obstetrics and Pediatrics Hospital	391	364
	**Quang Ninh province**			
		Quang Ninh General Hospital	922	675
		Bai Chay Hospital	819	476
		Quang Ninh Obstetrics and Pediatrics Hospital	478	290
**Center**				
	**Quang Tri province**			
		Trieu Phong District Health Center	271	203
	**Da Nang city**			
		Da Nang Oncology Hospital	555	134
**South**				
	**Ho Chi Minh city**			
		Tan Phu District Hospital	530	241
		Hospital District 2	812	318
		District 9 Health Center	170	102
		Thu Duc District Health Center	302	291
	**Can Tho city**			
		Can Tho University of Medicine and Pharmacy Hospital	424	191
Total			11,517	5209

Vietnam applied a nationwide lockdown measure April 1-15, 2020 [17], which was extended to April 22, 2020 [46,47]. In this study, HCWs took the online survey during the lockdown period. We used Google Forms to design and conduct the survey. The online survey links were sent to HCWs by researchers via email, Messenger, or Zalo. QR codes were also displayed in different departments of hospitals and health centers. It took about 15 minutes to complete the questionnaire. All survey questions were mandatory; therefore, there is no missing data in our study. All responses were exported to Google Sheets and saved on Google Drive. Finally, the data was coded, cleaned, and analyzed confidentially by researchers.

### Measures

#### Sociodemographics

HCWs reported their age (21-40 years versus 41-60 years), gender (woman versus man), marital status (never married versus ever married), ability to pay for medication (very or fairly difficult versus very or fairly easy), social status (low versus middle to high), profession (doctor, nurse, or other, the last of which included medical technicians, midwives, pharmacists, pharmacy technicians, administrative staff, catering staff, and cleaners), type of health care facility (second-line versus frontline, the latter of which includes the outpatient department, emergency department, quarantine and isolation areas, medical imaging and laboratory diagnosis department, and patient administration areas), and previous epidemic (eg, SARS, tuberculosis, influenza A) containment experience (no versus yes). Additionally, comorbidity was assessed using the Charlson comorbidity index items [48,49].

#### Health Literacy

A 12-item short-form health literacy questionnaire (HLS-SF12) was used. The questionnaire was validated and used in Asian countries [50], including Vietnam [51-54]. HCWs rated their perceived difficulty of items based on a 4-point Likert scale from 1=Very difficult to 4=Very easy. The health literacy index score was standardized to an unified metric from 0 to 50, with higher scores representing better health literacy [50,55].

#### eHealth Literacy

The widely used eHealth literacy scale (eHEALS) with 8 items was used to assess HCWs’ eHealth literacy skills [56]. The questionnaire was translated into Vietnamese by the researchers. The content was then validated by an expert panel (28 medical doctors, 7 nurses, and 9 nutrition and public health professionals). The expert panel suggested retaining the original rating and scoring system. HCWs rated their experiences using the internet for health information based on a 5-point Likert scale from 1=Strongly disagree to 5=Strongly agree. Scores range from 8 to 40, with high eHEALS scores representing better eHealth literacy.

#### Adherence to IPC Procedures

Participants reported the practices and activities performed related to COVID-19 IPC during health care interactions. The questionnaire was adapted from the interim guidance of the World Health Organization [57]. The IPC measures included the following: (1) wearing single-use gloves, (2) wearing a medical mask, (3) wearing a face shield or goggles/protective glasses, (4) wearing a disposable gown, (5) removing and replacing personal protective equipment (PPE) according to protocol, (6) performing hand hygiene before and after touching patients, (7) performing hand hygiene before and after performing any clean or aseptic procedure, (8) performing hand hygiene after exposure to body fluid, (9) performing hand hygiene after touching patients’ surroundings, and (10) decontaminating high-touch surfaces. HCWs quantified the frequency of performance of the above IPC procedures as recommended, where 0=Never, 1=Rarely (<20% of the time), 2=Occasionally (20% to <50% of the time), 3=Most of the time (50% to <95% of the time), and 4=Always (≥95% of the time). The total performance score of the 10 activities ranged from 0 to 40, with higher scores indicating better adherence to IPC procedures.

#### Lifestyle Changes

HCWs reported their current smoking (never/stopped/less versus unchanged or more), drinking (never/stopped/less versus unchanged or more), physical activity (never/stopped/less versus unchanged or more), and eating (less healthy versus unchanged or healthier) behaviors as compared with that before the pandemic [54].

#### Suspected COVID-19 Symptoms

HCWs were screened for suspected COVID-19 symptoms [58], including the common symptoms of fever, cough, and dyspnea, along with the less common symptoms of myalgia, fatigue, sputum production, confusion, headache, sore throat, rhinorrhea, chest pain, hemoptysis, diarrhea, and nausea/vomiting. HCWs with any of those symptoms were classified as having symptoms indicative of COVID-19.

### Ethical Consideration

The study was reviewed and approved by the Institutional Ethical Review Committee of Hanoi University of Public Health, Vietnam (Institutional Review Board number 133/2020/YTCC-HD3). The HCWs voluntarily took the survey.

### Data Analysis

#### Psychometric Properties of the eHEALS

##### Construct Validity

The construct validity of the eHEALS was examined using principal component analysis (PCA). An Kaiser-Meyer Olkin (KMO) value ≥0.6 was set to measure sampling adequacy and a Bartlett Test of Sphericity value <0.05 was set to determine the suitability of the data for PCA [59]. The oblique rotation (Promax) method was used.

##### Convergent Validity

The Spearman correlation was used to check the correlations between the eHEALS and its eight items.

##### Criterion Validity

The Pearson correlation between eHEALS and HLS-SF12 was estimated to provide evidence of criterion validity [60].

##### Floor and Ceiling Effects

The percentages of the lowest and highest score among HCWs were calculated. Minimal percentages (<15%) were recommended to eliminate floor and ceiling effects [61].

##### Reliability Analysis

The internal consistency of the eHEALS was estimated using the Cronbach α test. A Cronbach α value ≥.70 was designated as satisfactory reliability [62].

#### Health Literacy, eHealth Literacy, and Associated Factors

The distributions of HL and eHEALS scores in different categories of studied variables were explored using a one-way analysis of variance (ANOVA) test. In addition, bivariate and multivariate linear regression models were used to examine predictors of HL and eHEALS scores and to investigate the associations of HL and eHEALS scores with adherence to IPC measures. Next, bivariate and multivariate logistic regression models were used to examine the associations of HL and eHEALS scores with lifestyle changes and suspected COVID-19 symptoms. The factors that demonstrated associations with outcome variables at *P* values <.20 in the bivariate models were included in multivariate models [63]. To exclude colliders, which may cause multicollinearity, we checked the correlations between them using the Spearman correlation test. The representative factors were selected in the multivariate analysis. The regression coefficient (B), odds ratio (OR), and 95% confidence interval (95% CI) were reported appropriately.

Data were analyzed using IBM SPSS (Version 20.0; IBM Corp). The significance level was set at a *P* value <.05.

## Results

### Characteristics of HCWs

Table 2 indicates that, of 5209 HCWs, 905 (17.4%) were aged 41-60 years, 1714 (32.9%) were men, 3915 (75.2%) were ever married, 2458 (47.2%) could very or fairly easily pay for medication, 4506 (86.5%) had a middle or high social status, 2540 (48.8%) were nurses, 1453 (27.9%) were doctors, 2299 (44.1%) were frontline HCWs, 1999 (38.4%) had epidemic containment experience, 270 (5.2%) had comorbidities, 769 (14.8%) had had suspected COVID-19 symptoms, 5042 (96.8%) ate at an “unchanged or healthier” level, 228 (4.4%) smoked at an “unchanged or more” level, 234 (4.5%) drank at an “unchanged or more” level, and 3553 (68.2%) had an “unchanged or more” physical activity level during the pandemic. The mean (SD) scores of adherence to IPC measures, HL, and eHEALS were 30.6 (6.2), 36.2 (7.3), and 33.1 (4.8), respectively. The HL scores varied by age, gender, ability to pay for medication, social status, type of health care personnel, epidemic containment experience, BMI, suspected COVID-19 symptoms, dietary intake, smoking, and physical activity (*P*<.05). The eHEALS scores varied by gender, ability to pay for medication, type of health care personnel, epidemic containment experience, BMI, suspected COVID-19 symptoms, dietary intake, smoking, and physical activity (Table 2).

**Table 2:** Characteristics, health literacy, and eHealth literacy among health care workers.

Variables			Total (N=5209)		Health literacy				eHealth Literacy Scale		
			Participants, n (%)		Mean (SD)		*P* value^a^		Mean (SD)		*P* value^a^
**Age (years)**							.02		N/A^b^		.88
	21-40	4304 (82.6)		36.1 (7.2)		N/A		33.1 (4.7)		N/A	
	41-60	905 (17.4)		36.7 (7.5)		N/A		33.1 (5.0)		N/A	
**Gender**							<.001		N/A		<.001
	Women	3495 (67.1)		35.7 (7.0)		N/A		32.8 (4.5)		N/A	
	Men	1714 (32.9)		37.1 (7.8)		N/A		33.8 (5.3)		N/A	
**Marital status**							.04		N/A		.29
	Never married	1294 (24.8)		35.8 (7.0)		N/A		33.0 (4.8)		N/A	
	Ever married	3915 (75.2)		36.3 (7.4)		N/A		33.2 (4.7)		N/A	
**Ability to pay for medication**							<.001		N/A		<.001
	Very or fairly difficult	2751 (52.8)		35.3 (7.4)		N/A		32.8 (4.9)		N/A	
	Very or fairly easy	2458 (47.2)		37.2 (7.0)		N/A		33.5 (4.6)		N/A	
**Social status**							<.001		N/A		.27
	Low	703 (13.5)		35.0 (7.7)		N/A		32.9 (4.7)		N/A	
	Middle or high	4506 (86.5)		36.3 (7.2)		N/A		33.2 (4.8)		N/A	
**Type of health care personnel**							<.001		N/A		<.001
	Other	1216 (23.3)		35.7 (7.6)		N/A		33.1 (5.1)		N/A	
	Nurse	2540 (48.8)		35.7 (7.2)		N/A		32.7 (4.6)		N/A	
	Doctor	1453 (27.9)		37.3 (7.1)		N/A		33.9 (4.8)		N/A	
**Type of health care facility^c^**							.77		N/A		.46
	Non-frontline	2910 (55.9)		36.2 (7.4)		N/A		33.2 (4.7)			
	Frontline	2299 (44.1)		36.1 (7.1)		N/A		33.1 (4.8)		N/A	
**Epidemic containment experience**							<.001		N/A		<.001
	No	3210 (61.6)		35.4 (7.2)		N/A		32.9 (4.6)		N/A	
	Yes	1999 (38.4)		37.4 (7.3)		N/A		33.5 (5.0)		N/A	
**Comorbidity**							.38		N/A		.37
	None	4939 (94.8)		36.1 (7.3)		N/A		33.1 (4.8)		N/A	
	One or more	270 (5.2)		36.5 (7.6)		N/A		32.9 (5.0)		N/A	
**Suspected COVID-19 symptoms^d^**							<.001		N/A		<.001
	No	4440 (85.2)		36.4 (7.3)		N/A		33.3 (4.7)		N/A	
	Yes	769 (14.8)		34.7 (7.2)		N/A		32.3 (5.1)		N/A	
**Dietary intake^e^**							<.001		N/A		.002
	Eat less healthy	167 (3.2)		34.2 (6.9)		N/A		32.0 (5.9)		N/A	
	Unchanged or healthier	5042 (96.8)		36.2 (7.3)		N/A		33.2 (4.7)		N/A	
**Smoking tobacco^f^**							.046		N/A		.03
	Never, stopped, or less	4981 (95.6)		36.1 (7.2)		N/A		33.1 (4.7)		N/A	
	Unchanged or more	228 (4.4)		37.1 (8.6)		N/A		33.8 (5.5)		N/A	
**Drinking alcohol^f^**							.18		N/A		.53
	Never, stopped, or less	4975 (95.5)		36.1 (7.2)		N/A		33.1 (4.7)		N/A	
	Unchanged or more	234 (4.5)		36.8 (8.3)		N/A		33.3 (5.7)		N/A	
**Physical activity^f^**							<.001		N/A		<.001
	Never, stopped, or less	1656 (31.8)		35.2 (7.6)		N/A		32.5 (5.2)		N/A	
	Unchanged or more	3553 (68.2)		36.6 (7.1)		N/A		33.4 (4.6)		N/A	
Adherence to infection prevention and control measures, mean (SD)			30.6 (6.2)		N/A		N/A		N/A		N/A
Health literacy index, mean (SD)			36.2 (7.3)		N/A		N/A		N/A		N/A
eHealth Literacy Scale score, mean (SD)			33.1 (4.8)		N/A		N/A		N/A		N/A

^a^*P* value: result of one-way analysis of variance (ANOVA) test.

^b^N/A: not applicable.

^c^Frontline areas include the outpatient department, emergency department, isolation areas, imaging and laboratory diagnosis department, and patient administration areas.

^d^Suspected COVID-19 symptoms include common symptoms (fever, cough, dyspnea) and less common symptoms (myalgia, fatigue, sputum production, confusion, headache, sore throat, rhinorrhea, chest pain, hemoptysis, diarrhea, and nausea/vomiting).

^f^Health care workers were asked whether their lifestyle behaviors got worse, better, or were unchanged during the COVID-19 pandemic as compared to before the pandemic.

### Psychometric Properties of eHealth Literacy

As shown in Table 3, the KMO value of the overall scale was 0.93 and the values of its 8 items ranged from 0.92 to 0.95. The Bartlett Test of Sphericity value was <0.001, which is a satisfactory level. In addition, the average communality value of 0.76 was satisfactory, demonstrating the accuracy of the approach [59]. Overall, the 8 items of the eHEALS were strongly loaded on one component and explained 76.34% of the scale variance. The factor loading values of 8 items were ranked from 0.78 to 0.92, as shown in Table 3. The correlations between each item and the scale range from 0.80 to 0.84, indicating satisfactory convergent validity [50,51]. In addition, the correlation between eHEALS and HL scores was moderate (ρ=0.42), providing evidence of criterion validity [60]. Furthermore, the Cronbachα value of .95 indicated a high level of internal consistency. There was no significant floor effect, with 0.70% of participants having the lowest potential response. A marginal ceiling effect was found, with 16.1% of participants having the highest potential response (slightly higher than the recommended percentage of <15%; Table 3).

**Table 3:** Construct, convergent, and criterion validity, internal consistency, and floor and ceiling effects of the 8-item eHealth Literacy Scale (N=5209).

Construct validity, factor loadings		Values
**eHealth Literacy scale items**		
	I know what health resources are available on the internet	0.85
	I know where to find helpful health resources on the internet	0.90
	I know how to find helpful health resources on the internet	0.92
	I know how to use the internet to answer my questions about health	0.90
	I know how to use the health information I find on the internet to help me	0.92
	I have the skills I need to evaluate the health resources I find on the internet	0.89
	I have the skills needed to tell high-quality health resources from low-quality health resources on the internet	0.82
	I feel confident in using information from the internet to make health decisions	0.78
Percentage of variance, %		76.34
Item-scale convergent validity, mean of ρ^a^ (range)		0.83 (0.80-0.84)
Criterion validity, correlation with health literacy, ρ^b^		0.42
Internal consistency, Cronbach α		.95
Floor effect, %		0.70
Ceiling effect, %		16.10

^a^ρ: Spearman correlation coefficient.

^b^ρ: Pearson correlation coefficient.

### Determinants of HL and eHealth Literacy

The week correlations among independent variables (ρ <0.30) suggest that there is no collider which might affect the results (Table 1 in Multimedia Appendix 1). Results of the multivariate analysis shown in Table 4 indicated that HCWs with higher HL scores were men (unstandardized regression coefficient [B]=1.01, 95% CI 0.57-1.45, *P*<.001), those with a very or fairly easy ability to pay for medication (B=1.65, 95% CI 1.25-2.05, *P*<.001), those with a middle or high social status (B=0.586, 95% CI 0.003-1.169, *P*=.049), doctors (B=1.29, 95% CI 0.73-1.84, *P*<.001), and those with epidemic containment experience (B=1.96, 95% CI 1.56-2.37, *P*<.001), as compared to their counterparts, respectively.

HCWs with higher eHEALS scores were men (B=0.72, 95% CI 0.43-1.00, *P*<.001), those with a very or fairly easy ability to pay for medication (B=0.60, 95% CI 0.34-0.86, *P*<.001), doctors (B=0.56, 95% CI 0.20-0.93, *P*=.003), and those with epidemic containment experience (B=0.64, 95% CI 0.38-0.91, *P*<.001), as compared to their counterparts, respectively (Table 4).

**Table 4:** Determinants of health literacy and eHealth literacy among health care workers (N=5209).

Variables			Health literacy								eHealth literacy						
			Bivariate				Multivariate				Bivariate				Multivariate		
			B^a^ (95% CI)		*P* value		B (95% CI)		*P* value		B (95% CI)		*P* value		B (95% CI)		*P* value
**Age (years)**																	
	21-40	Reference		N/A^b^		Reference		N/A		Reference		N/A		Reference		N/A	
	41-60	0.62 (0.10 to 1.14)		.02		–0.05 (–0.58 to 0.49)		.86		–0.03 (–0.37 to 0.31)		.88		–0.30 (–0.65 to 0.04)		.08	
**Gender**																	
	Female	Reference		N/A		Reference		N/A		Reference		N/A		Reference		N/A	
	Male	1.36 (0.94 to 1.78)		<.001		1.01 (0.57 to 1.45)		<.001		0.99 (0.72 to 1.27)		<.001		0.72 (0.43 to 1.00)		<.001	
**Marital status**																	
	Never married	Reference		N/A		Reference		N/A		Reference		N/A		N/A		N/A	
	Ever married	0.47 (0.01 to 0.93)		.04		0.34 (–0.12 to 0.81)		.15		0.16 (–0.14 to 0.46)		.29		N/A		N/A	
**Ability to pay for medication**																	
	Very or fairly difficult	Reference		N/A		Reference		N/A		Reference		N/A		Reference		N/A	
	Very or fairly easy	1.9 (1.51 to 2.29)		<.001		1.65 (1.25 to 2.05)		<.001		0.72 (0.46 to 0.98)		<.001		0.60 (0.34 to 0.86)		<.001	
**Social status**																	
	Low	Reference		N/A		Reference		N/A		Reference		N/A		N/A		N/A	
	Middle or high	1.29 (0.72 to 1.87)		<.001		0.586 (0.003 to 1.169)		.049		0.22 (–0.16 to 0.59)		.27		N/A		N/A	
**Type of health care personnel**																	
	Other	Reference		N/A		Reference		N/A		Reference		N/A		Reference		N/A	
	Nurse	0.09 (–0.41 to 0.58)		.73		0.18 (–0.32 to 0.68)		.48		–0.40 (–0.72 to –0.07)		.02		–0.32 (–0.65 to 0.01)		.06	
	Doctor	1.68 (1.13 to 2.24)		<.001		1.29 (0.73 to 1.84)		<.001		0.76 (0.39 to 1.12)		<.001		0.56 (0.20 to 0.93)		.003	
**Type of health care facility^c^**																	
	Non-frontline	Reference		N/A		N/A		N/A		Reference		N/A		N/A		N/A	
	Frontline	–0.06 (–0.46 to 0.34)		.77		N/A		N/A		–0.10 (–0.36 to 0.16)		.46		N/A		N/A	
**Epidemic containment experience**																	
	No	Reference		N/A		Reference		N/A		Reference		N/A		Reference		N/A	
	Yes	1.95 (1.54 to 2.35)		<.001		1.96 (1.56 to 2.37)		<.001		0.55 (0.29 to 0.82)		<.001		0.64 (0.38 to 0.91)		<.001	
**Comorbidity**																	
	None	Reference		N/A		Reference		N/A		Reference		N/A		N/A		N/A	
	One or more	0.40 (–0.49 to 1.29)		.378		N/A		N/A		–0.27 (–0.85 to 0.32)		.369		N/A		N/A	

^a^B: unstandardized regression coefficient.

^b^N/A: not applicable.

^c^Frontline areas are the outpatient department, emergency department, isolation areas, imaging and laboratory diagnosis department, and patient administration areas.

### Association Between HL and eHealth Literacy and Adherence to IPC Procedures

According to the results of a multivariate linear regression analysis (Table 5), higher HL (B=0.13, 95% CI 0.10-0.15, *P*<.001) and eHEALS scores (B=0.22, 95% CI 0.19-0.26, *P*<.001) were found to be associated with better adherence to IPC measures after adjusting for age, gender, ability to pay for medication, social status, profession, type of health care facility, epidemic containment experience, and comorbidities. These adjusted factors showed the associations with adherence to IPC measures at *P*<.20 (Table 2 in Multimedia Appendix 1).

**Table 5:** Associations of health literacy and eHealth literacy with adherence to infection prevention and control measures, lifestyle changes, and suspected COVID-19 symptoms among health care workers (N=5209).

Variables		Adherence to IPC measures^a^		Dietary intake^b^		Smoking tobacco^c^		Drinking alcohol^d^		Physical activity^e^		Suspected COVID-19 symptoms^f^	
		B^g^ (95% CI)	*P* value	OR^h^ (95% CI)	*P* value	OR (95% CI)	*P* value	OR (95% CI)	*P* value	OR (95% CI)	*P* value	OR (95% CI)	*P* value
**Health literacy, 1-score increment**													
	Bivariate model	0.13 (0.11-0.15)	<.001	1.04 (1.02-1.06)	<.001	1.02 (1.00-1.04)	.046	1.01 (0.99-1.03)	.18	1.03 (1.02-1.04)	<.001	0.97 (0.96-0.98)	<.001
	Multivariate model	0.13 (0.10-0.15)	<.001	1.04 (1.01-1.06)	.001	1.01 (0.99-1.03)	.34	1.01 (0.99-1.02)	.47	1.03 (1.02-1.03)	<.001	0.97 (0.96-0.98)	<.001
**eHealth Literacy Scale, 1-score increment**													
	Bivariate model	0.22 (0.18-0.25)	<.001	1.04 (1.02-1.07)	.002	1.03 (1-1.07)	.03	1.01 (0.98-1.04)	.53	1.04 (1.03-1.05)	<.001	0.96 (0.95-0.98)	<.001
	Multivariate model	0.22 (0.19-0.26)	<.001	1.04 (1.02-1.07)	.002	1.01 (0.99-1.04)	.30	0.99 (0.97-1.02)	.69	1.04 (1.03-1.05)	<.001	0.96 (0.95-0.98)	<.001

^a^Adherence to infection prevention and control procedures; adjusted for age, gender, ability to pay for medication, social status, type of health care personnel, type of health care facility, epidemic containment experience, and comorbidity in the multivariate model.

^b^Adjusted for age, gender, marital status, ability to pay for medication, and social status in the multivariate model. The reference group is “less healthy diet” and the test group is “unchanged or healthier diet.”

^c^Adjusted for age, gender, marital status, social status, and type of health care personnel in the multivariate model. The reference group is “never, stopped, or less smoking,” and the test group is “unchanged or more smoking.”

^d^Adjusted for age, gender, social status, type of health care personnel, and epidemic containment experience in the multivariate model. The reference group is “never, stopped, or less drinking,” and the test group is “unchanged or more drinking.”

^e^Adjusted for age, gender, ability to pay for medication, social status, type of health care personnel, type of health care facility, and epidemic containment experience in the multivariate model. The reference group is “never, stopped, or less physical activity,” and the test group is “unchanged or more physical activity.”

^f^Suspected COVID-19 symptoms include common symptoms (fever, cough, dyspnea) and less common symptoms (myalgia, fatigue, sputum production, confusion, headache, sore throat, rhinorrhea, chest pain, hemoptysis, diarrhea, and nausea/vomiting). Adjusted for age, gender, marital status, ability to pay for medications, social status, type of health care personnel, and comorbidity in the multivariate model.

^g^B: unstandardized regression coefficient.

^h^OR: odds ratio.

### Association Between HL and eHealth Literacy and Lifestyle Changes

The results of the multivariate logistic regression analysis shown in Table 5 indicated that HCWs with higher HL (OR 1.04, 95% CI 1.01-1.06, *P*=.001) and higher eHEALS scores (OR 1.04, 95% CI 1.02-1.07, *P*=.002) had a higher likelihood of eating at an “unchanged or healthier” level after being adjusted for age, gender, marital status, ability to pay for medication, and social status. These adjusted factors had associations with eating behavior changes at *P*<.20 (Table 2 in Multimedia Appendix 1). In addition, HCWs with higher HL scores (OR 1.03, 95% CI 1.02-1.03, *P*<.001) and higher eHEALS scores (OR 1.04, 95% CI 1.03-1.05, *P*<.001) had a higher likelihood of doing physical activity at an “unchanged or more” level after adjusting for age, gender, ability to pay for medication, social status, profession, type of health care facility, and epidemic containment experience. These adjusted factors had associations with physical activity at *P*<.20 (Table 2 in Multimedia Appendix 1). No association was found between HL and eHEALS scores and smoking or drinking behaviors.

### Associations of HL and eHealth Literacy With Suspected COVID-19 Symptoms

As shown in Table 5, HCWs with higher HL scores (OR 0.97, 95% CI 0.96-0.98, *P*<.001) and higher eHEALS scores (OR 0.96, 95% CI 0.95-0.98, *P*<.001) had a lower likelihood of having suspected COVID-19 symptoms after adjusting for age, gender, marital status, ability to pay for medication, social status, profession, and comorbidities. These adjusted factors had associations with suspected COVID-19 symptoms at *P*<.20 (Table 2 in Multimedia Appendix 1).

## Discussion

### Principal Findings

The eHEALS questionnaire was found to be valid and reliable for assessing the eHealth literacy of HCWs. The tool was found to have satisfactory construct validity, convergent validity, criterion validity, and reliability. There was no flooring effect. A marginal ceiling effect was found. A high percentage of participants with the highest possible score was also observed in a previous study that validated the digital health literacy scale [64]. This could be because HCWs have been recognized as a group that has a higher standard of health knowledge and skills than the average person. Therefore, it is reasonable that many participants had a higher or maximum score.

In this study, male HCWs had higher HL and eHEALS scores. This finding is inconsistent with a previous study conducted among the general population in Europe [65], in which men had lower HL scores than women. Another study conducted among the general population in Taiwan showed that men also had lower scores of HL and digital health diet literacy than women [66]. In addition, eHealth literacy was found to be higher in women than men in the general population in Hungary [67] and in health care professionals in Ethiopia [68]. This inconsistency could be explained by the fact that although the total HCW population had fewer males (32.9% versus 67.1% female), the proportion of male doctors (797/1453 or 54.9%) was higher than that of female doctors (656/1453 or 45.1%).

Doctors had higher HL and eHEALS scores than other HCWs in this study. Similarly, HCWs with epidemic containment experience had higher HL and eHEALS scores in this study. Among HCWs, doctors receive longer professional training and they have been recognized as the group with the highest level of ability to find, understand, justify, and use health-related information. In addition, they are involved in educating and counseling other HCWs and patients [69]. HCWs who were involved in containing previous outbreaks or epidemics logically had better health knowledge and skills that made their HL and eHEALS scores higher. HL was found to be a strategic approach for improving communication between patients and HCWs [41], further contributing to the containment of the pandemic and its consequences [45].

HCWs with a better ability to pay for medication had higher HL and eHEALS scores. A positive association between the ability to pay for medication was prominently found in many previous studies [51,52,66,70,71]. In addition, HCWs with middle or high social status had higher HL scores. This was similar to the findings of previous studies conducted in Asia [51,52,66,70-72] and Europe [65]. This implies that socioeconomic factors are independent indictors that should be taken into consideration in intervention development to improve HL and eHEALS scores.

In this study, HCWs with higher HL or eHEALS scores had better adherence to IPC procedures. This is the first study investigating these associations. In the literature, higher HL scores were found to be associated with better adherence to therapies among people with chronic diseases [73-76]. Receiving COVID-19–related information online during the pandemic could be beneficial for various preventive behaviors [77]. Therefore, more efforts are needed to increase the distribution of accurate information and encourage appropriate behaviors [78]. HCWs should be aware of and strictly adhere to the guidelines for IPC to contain the COVID-19 pandemic.

Our study found that HCWs with higher HL and eHEALS scores had a lower likelihood of suspected COVID-19 symptoms. It has been noted that individuals with higher HL scores had better health status [71,79] and well-being [80,81]. This could be further explained by the fact that higher HL and eHEALS scores were associated with healthier eating and physical activity behaviors in this study. In a previous study, we also found that medical and nursing students with higher digital healthy diet literacy had healthier eating behaviors during the pandemic [82]. In addition, eHealth literacy was found to be associated with key issues related to seeking out exercise regime information and maintaining a healthy diet [83]. In addition, it has been reported that people with higher HL scores had healthier behaviors (eg, exercise, balanced diet) [66,84], further protecting and improving their health and well-being [85].

This study has some limitations. First, the study was conducted online and the suspected COVID-19 symptoms were self-reported; therefore, we cannot confirm COVID-19 cases and exclude them from our study. Fortunately, there were no new confirmed cases during the data collection period [86]. Second, during the lockdown period, people were ordered to stay at home and only go out to buy food or medicine, for emergencies, or to work at businesses producing and providing essential goods and services. This may cause a measurement bias in the application domain of HL, as the questionnaire cannot distinguish between an individual’s ability and restrictions during lockdown (eg, on questions such as “on a scale from very easy to very difficult, how easy would you say it is to join a sports club or exercise class if you want to?”). Third, a cross-sectional design was used, which cannot provide evidence of the causal relationships among studied variables. Furthermore, the convenient sampling technique used may limit the generalizability of the findings. In addition, the differences between some categories were not examined due to small sample sizes (eg, widowed/separated/divorced participants [140/5209, 2.7%] or those with high social status 63/5209, 1.2%]). However, we surveyed a relatively large sample of HCWs from 15 hospitals and health centers across Vietnam, which can help with exploring associations in future research and provides evidence for potential interventions.

### Conclusions

The eHEALS questionnaire is a valid and reliable tool for assessing eHealth literacy among HCWs. HL and eHealth literacy were significantly higher in men, those with better ability to pay for medication, doctors, and those with previous epidemic containment experience. Both HL and eHealth literacy were associated with better adherence to IPC procedures, healthier lifestyles (eg, healthier eating behavior and more physical activity during the pandemic), and a lower likelihood of having suspected COVID-19 symptoms. Integrative and multidisciplinary approaches are required to improve HCWs’ HL and eHealth literacy, which could help improve adherence to IPC measures, promote healthy behaviors, and protect the health of HCWs. This would further contribute to containing the COVID-19 pandemic and minimizing its consequences.

## Appendix

Multimedia Appendix 1 Supplementary data.

## Abbreviations

B: unstandardized regression coefficient
eHEALS: eHealth Literacy Scale
HCWs: health care workers
HL: health literacy
HLS-SF12: 12-item short-form health literacy questionnaire
IPC: infection prevention and control
KMO: Kaiser-Meyer Olkin Measure
PCA: principal component analysis
PPE: personal protective equipment
WHO: World Health Organization
