# Supplementary material for: Health Literacy, eHealth Literacy, Adherence to Infection Prevention and Control Procedures, Lifestyle Changes, and Suspected COVID-19 Symptoms Among Health Care Workers During Lockdown: Online Survey
Source: J Med Internet Res. 2020 Nov 12;22(11):e22894. doi: 10.2196/22894 (PMC7674138; doi:10.2196/22894)
Supplement: Multimedia Appendix 1 [file jmir_v22i11e22894_app1.docx]

**Multimedia Appendix 1**

**Text 1. Online survey questionnaires for health care workers in Vietnam.** 2

**Table 1. Spearman’s correlations among independent variables (N=5209).** 9

**Table 2. Determinants of adherence to infection prevention and control measures, lifestyle changes, and suspected COVID-19 symptoms among health care workers via bivariate linear or logistic regression models (N=5209).** 10

# **Text 1. Online survey questionnaires for health care workers in Vietnam.**

Start of Block: Informed Consent

Q0. **DESCRIPTION:** You are invited to participate in a survey related to health literacy, adherence to infection prevention and control among healthcare workers in Vietnam during global COVID-19 pandemic. You will be presented with questions related to social demographics, and your practice and activities related to infection prevention and control. The findings will serve as the evidence for appropriate interventions in hospitals, health centers, in order to prevent and control emerged COVID-19 in Vietnam and other countries. You will be asked to select options in multiple choice questions or type answers into text boxes.

Your participation will take approximately 10-15 minutes.

There are no foreseeable risks associated with this study. There are no direct benefits to you from participating in this study. However, the information you provide may help improve public health efforts with regards to the new coronavirus and, thus, reduce harm from this epidemic.

If you have read this form and have decided to participate in this survey, please understand your participation is voluntary and you have the right to withdraw your consent or discontinue participation at any time without penalty or loss of benefits to which you are otherwise entitled. The alternative is not to participate. You have the right to refuse to answer particular questions. The results of this research study may be presented at scientific or professional meetings or published in scientific journals. Your individual privacy will be maintained in all published and written data resulting from the study.

If you agree to participate in this research, please select “I consent, begin the study” below.

- (1) I consent, begin the study
- (2) I do not consent; I do not wish to participate

End of Block: Informed Consent

Start of Block: Socio-demographics

**Q1.1 Which hospitals or health centers are you working for?**

- Military Hospital 103
- E Hospital
- General Hospital of Agricultural
- Thai Nguyen National Hospital
- Bac Ninh Obstetric and Pediatric Hospital
- Quang Ninh General Hospital
- Bai Chay Hospital
- Quang Ninh Obstetric and Pediatric Hospital
- Trieu Phong District Health Centers
- Da Nang Oncology Hospital
- Tan Phu District Hospital
- Hospital District 2
- District 9 Health Center
- Thu Duc District Health Center
- Can Tho University of Medicine and Pharmacy Hospital

**Q1.2** What is your type of health care personnel?

- Doctors
- Nurses
- Technicians
- Other (specify): ______________

**Q1.3** Which type of health care facility are you working now?

- Outpatient department
- Emergency department
- Quarantine, isolation areas
- Inpatient department
- Medical imaging and laboratory diagnosis department
- Patient administration areas which have exposed patients.
- Patient administration areas which have not exposed patients.

**Q1.4** Previous epidemics (e.g. SARS, Tuberculosis, influenza A) containment experiences

- Yes
- No
- Don’t known

**Q1.5** When did you born? *year* _ _ _ _

**Q1.6** What is your gender?

- Male
- Female

**Q1.7** What is your marital status?

- Single
- Married
- Separated/divorced/widowed

**Q1.8** How could you tell about your ability to pay for medication?

- Very difficult
- Fairly difficult
- Fairly easy
- Very easy

**Q1.9** If you self-assess about your social status, which level you are?

- Low
- Moderate
- High

End of Block: Socio-demographics

Start of Block: the suspected COVID-19 symptoms

Q2. Which of the following symptoms do you have? *(Select all options that you have)*

- Fever
- Cough
- Dyspnea
- Myalgia
- Fatigue
- Sputum production
- Confusion
- Headache
- Sore throat
- Rhinorrhea
- Chest pain
- Hemoptysis
- Diarrhea
- Nausea/vomiting
- Other (specify): ­­­­­­______________________
- Do not have any suspected COVID-19 symptoms

End of Block: the suspected COVID-19 symptoms

Start of Block: Adherence to infection prevention and control procedures

**Q3.** Those questions below ask about the practice and activities performed on COVID-19 infection prevention and control (IPC) during the health care interactions. In the process of contacting and taking care of the patient, what level of compliance did you do?
  *Please select the frequency with which they performed the IPC procedures, as recommended from 0=never, 1= rarely (less than 20% of the time), 2= occasionally (20% to under 50% of the time), 3= most of the time (50% under 95% of the time), 4= always (≥ 95% of the time) for each question.*

|  | During the health care interactions, did you… | Never  ▼  0 | Rarely  ▼  1 | Occasionally  ▼  2 | Most of the time  ▼  3 | Always  ▼  4 |
| --- | --- | --- | --- | --- | --- | --- |
| **3.1** | **… wear personal protective equipment (PPE)** |  |  |  |  |  |
|  | 1. single-used gloves | ○ | ○ | ○ | ○ | ○ |
|  | 1. medical mask | ○ | ○ | ○ | ○ | ○ |
|  | 1. face shield or goggles/protective glasses | ○ | ○ | ○ | ○ | ○ |
|  | 1. disposable gown | ○ | ○ | ○ | ○ | ○ |
| **3.2** | **…remove and replace the PPE according to protocol** | ○ | ○ | ○ | ○ | ○ |
| **3.3** | **…perform hand hygiene** |  |  |  |  |  |
|  | 1. before and after touching patients | ○ | ○ | ○ | ○ | ○ |
|  | 1. before and after performing any clean or aseptic procedure | ○ | ○ | ○ | ○ | ○ |
|  | 1. after exposure to body fluid | ○ | ○ | ○ | ○ | ○ |
|  | 1. after touching patients’ surroundings | ○ | ○ | ○ | ○ | ○ |
| **3.4** | **…decontaminate high-touch surfaces (at least 3 times/day)** | ○ | ○ | ○ | ○ | ○ |

End of Block: Adherence to infection prevention and control

**Start of Block: Health literacy**

**Q4.** Those questions below ask about your perceived difficulty of each item based on 4-point Likert scales: *1= very difficult, 2= difficult, 3= easy, and 4= very easy. Please rate your experiences for each question.*

| On a scale from very easy to very difficult, how easy would you say it is to: | | Very difficult  ▼  1 | Difficult  ▼  2 | Easy  ▼  3 | very easy  ▼  4 |
| --- | --- | --- | --- | --- | --- |
|  | …find information on treatments of illnesses that concern you? | ○ | ○ | ○ | ○ |
|  | …understand the leaflets that come with your medicine? | ○ | ○ | ○ | ○ |
|  | …judge the advantages and disadvantages of different treatment options? | ○ | ○ | ○ | ○ |
|  | …call an ambulance in an emergency? | ○ | ○ | ○ | ○ |
|  | …find information on how to manage mental health problems like stress or depression? | ○ | ○ | ○ | ○ |
|  | …understand why you need health screenings (such as breast exam, blood sugar test, blood pressure)? | ○ | ○ | ○ | ○ |
|  | …judge which vaccinations you may need? | ○ | ○ | ○ | ○ |
|  | …decide how you can protect yourself from illness based on advice from family and friends? | ○ | ○ | ○ | ○ |
|  | …find out about activities (such as meditation, exercise, walking, Pilates etc.) that are good for your mental well-being? | ○ | ○ | ○ | ○ |
|  | …understand information in the media (such as Internet, newspaper, magazines) on how to get healthier? | ○ | ○ | ○ | ○ |
|  | …judge which everyday behavior (such as drinking and eating habits, exercise etc.) is related to your health? | ○ | ○ | ○ | ○ |
|  | … join a sports club or exercise class if you want to? | ○ | ○ | ○ | ○ |

End of Block: Health literacy

Start of Block: eHealth literacy

**Q5.** Those questions below ask about your experiences using the Internet for health information based on a 5-points Likert scale: *1=strongly disagree, 2=disagree, 3=neutral, 4=agree, and 5=strongly agree. Please rate your experiences for each question.*

| Your experiences using the Internet for health information | | Strongly disagree  ▼  1 | Disagree  ▼  2 | Neutral  ▼  3 | Agree  ▼  4 | Strongly agree  ▼  5 |
| --- | --- | --- | --- | --- | --- | --- |
|  | I know how to find helpful health resources on the Internet | ○ | ○ | ○ | ○ | ○ |
|  | I know how to use the Internet to answer my health questions | ○ | ○ | ○ | ○ | ○ |
|  | I know what health resources are available on the Internet | ○ | ○ | ○ | ○ | ○ |
|  | I know where to find helpful health resources on the Internet | ○ | ○ | ○ | ○ | ○ |
|  | I know how to use the health information I find on the Internet to help me | ○ | ○ | ○ | ○ | ○ |
|  | I have the skills I need to evaluate the health resources I find on the Internet | ○ | ○ | ○ | ○ | ○ |
|  | I can tell high quality from low quality health resources on the Internet | ○ | ○ | ○ | ○ | ○ |
|  | I feel confident in using information from the Internet to make health decisions | ○ | ○ | ○ | ○ | ○ |

End of Block: E-health literacy

**Start of Block: Lifestyle changes (dietary eating, smoking cigarettes, drinking alcohol, physical activities)**

**Q6.** Could you tell us about your current status of dietary intake as compared with that before the pandemic?

|  |  | Less healthy  ▼  3 | Unchanged  ▼  4 | Healthier  ▼  5 |
| --- | --- | --- | --- | --- |
| **6.1** | Dietary intake | ○ | ○ | ○ |

**Q7.** Could you tell us about your current status of smoking cigarettes, drinking alcohol, physical activities as compared with those before the pandemic?

|  |  | Never  ▼  1 | Stopped  ▼  2 | Less  ▼  3 | Unchanged  ▼  4 | More  ▼  5 |
| --- | --- | --- | --- | --- | --- | --- |
| **7.1** | Smoking? | ○ | ○ | ○ | ○ | ○ |
| **7.2** | Drinking? | ○ | ○ | ○ | ○ | ○ |
| **7.3** | Physical activities? | ○ | ○ | ○ | ○ | ○ |

End of Block: Lifestyle changes (smoking, alcohol consumption, physical activities)

**Start of Block: Thank you**

End of Block: Thank you

# **Table 1. Spearman’s correlations among independent variables (N=5209).**

|  | Age | Gender | Marital status | Ability to pay for medication | Social status | Health care personnel | Health care facility | Epidemic containment experience |
| --- | --- | --- | --- | --- | --- | --- | --- | --- |
| Gender | 0.032 |  |  |  |  |  |  |  |
| Marital status | 0.239 | -0.020 |  |  |  |  |  |  |
| Ability to pay for medication | 0.039 | 0.030 | -0.002 |  |  |  |  |  |
| Social status | 0.061 | -0.043 | 0.047 | 0.192 |  |  |  |  |
| Health care personnel | -0.016 | -0.165 | 0.056 | -0.096 | -0.119 |  |  |  |
| Health care facility | 0.059 | 0.006 | 0.026 | 0.003 | 0.004 | 0.139 |  |  |
| Epidemic containment experience | 0.129 | 0.011 | 0.089 | 0.000 | 0.037 | 0.020 | 0.099 |  |
| Comorbidity | 0.126 | 0.017 | 0.052 | -0.008 | -0.029 | -0.010 | 0.028 | 0.022 |

# **Table 2. Determinants of adherence to infection prevention and control measures, lifestyle changes, and suspected COVID-19 symptoms among health care workers via bivariate linear or logistic regression models (N=5209).**

| Variables | AIPC^a^ |  | Dietary intake^b^ |  | Smoking tobacco^c^ |  | Drinking alcohol^d^ |  | Physical activity^e^ |  | S-COVID-19-S^f^ |  |
| --- | --- | --- | --- | --- | --- | --- | --- | --- | --- | --- | --- | --- |
|  | B^g^ (95%CI)^h^ | *P* value | OR^i^ (95%CI) | *P* value | OR^i^ (95%CI) | *P* value | OR^i^ (95%CI) | *P* value | OR^i^ (95%CI) | *P* value | OR^i^ (95%CI) | *P* value |
| **Age, year** |  |  |  |  |  |  |  |  |  |  |  |  |
| 21-40 | Reference |  | Reference |  | Reference |  | Reference |  | Reference |  | Reference |  |
| 41-60 | 0.74 (0.29 to 1.18) | .001 | 2.78 (1.54-5.02) | .001 | 1.43 (1.04-1.97) | .028 | 0.98 (0.69-1.39) | .908 | 1.05 (0.9-1.22) | .545 | 0.99 (0.81-1.22) | .950 |
| **Gender** |  |  |  |  |  |  |  |  |  |  |  |  |
| Women | Reference |  | Reference |  | Reference |  | Reference |  | Reference |  | Reference |  |
| Men | -0.30 (-0.66 to 0.05) | .096 | 0.45 (0.33-0.61) | <.001 | 4.66 (3.51-6.19) | <.001 | 2.88 (2.21-3.76) | <.001 | 1.05 (0.93-1.19) | .451 | 1.08 (0.92-1.27) | .362 |
| **Marital status** |  |  |  |  |  |  |  |  |  |  |  |  |
| Never married | Reference |  | Reference |  | Reference |  | Reference |  | Reference |  | Reference |  |
| Ever married | -0.02 (-0.41 to 0.37) | .917 | 2.08 (1.52-2.86) | <.001 | 1.36 (0.98-1.9) | .069 | 0.83 (0.62-1.12) | .224 | 1.04 (0.91-1.19) | .600 | 0.87 (0.73-1.04) | .125 |
| **Ability to pay for medication** |  |  |  |  |  |  |  |  |  |  |  |  |
| Very or fairly difficult | Reference |  | Reference |  | Reference |  | Reference |  | Reference |  | Reference |  |
| Very or fairly easy | 0.23 (-0.11 to 0.56) | .188 | 2.02 (1.45-2.81) | <.001 | 0.99 (0.76-1.29) | .936 | 0.99 (0.76-1.29) | .955 | 1.21 (1.08-1.37) | .001 | 0.7 (0.6-0.82) | <.001 |
| **Social status** |  |  |  |  |  |  |  |  |  |  |  |  |
| Low | Reference |  | Reference |  | Reference |  | Reference |  | Reference |  | Reference |  |
| Middle or high | 0.91 (0.42 to 1.40) | <.001 | 1.8 (1.24-2.63) | .002 | 0.66 (0.47-0.92) | .016 | 0.66 (0.47-0.92) | .016 | 1.15 (0.97-1.36) | .107 | 0.58 (0.48-0.71) | <.001 |
| **Type of health care personnel** |  |  |  |  |  |  |  |  |  |  |  |  |
| Others | Reference |  | Reference |  | Reference |  | Reference |  | Reference |  | Reference |  |
| Nurse | -0.56 (-0.99 to -0.14) | .009 | 1.03 (0.69-1.54) | .879 | 0.61 (0.44-0.85) | .004 | 0.58 (0.40-0.82) | .002 | 1.10 (0.95-1.27) | .216 | 1.18 (0.97-1.44) | .090 |
| Doctor | -1.20 (-1.67 to -0.73) | <.001 | 0.80 (0.52-1.22) | .296 | 1.11 (0.79-1.55) | .548 | 1.62 (1.16-2.25) | .004 | 1.12 (0.95-1.32) | .168 | 0.97 (0.78-1.21) | .807 |
| **Type of health care facility^j^** |  |  |  |  |  |  |  |  |  |  |  |  |
| Non-front-line | Reference |  | Reference |  | Reference |  | Reference |  | Reference |  | Reference |  |
| Front-line | 1.47 (1.13 to 1.80) | <.001 | 0.97 (0.71-1.32) | .838 | 1.08 (0.83-1.42) | .551 | 0.91 (0.70-1.18) | .477 | 1.08 (0.96-1.22) | .190 | 0.99 (0.85-1.16) | .912 |
| **Epidemic containment experience** |  |  |  |  |  |  |  |  |  |  |  |  |
| No | Reference |  | Reference |  | Reference |  | Reference |  | Reference |  | Reference |  |
| Yes | 1.48 (1.13 to 1.82) | <.001 | 0.98 (0.71-1.34) | .883 | 1.07 (0.82-1.4) | .626 | 0.72 (0.54-0.95) | .021 | 1.18 (1.05-1.33) | .006 | 0.93 (0.79-1.09) | .372 |
| **Comorbidity** |  |  |  |  |  |  |  |  |  |  |  |  |
| None | Reference |  | Reference |  | Reference |  | Reference |  | Reference |  | Reference |  |
| One or more | -1.44 (-2.20 to -0.68) | <.001 | 0.70 (0.38-1.27) | .238 | 1.31 (0.76-2.24) | .332 | 1.17 (0.67-2.04) | .573 | 1.09 (0.84-1.43) | .516 | 3.96 (3.05-5.13) | <.001 |
| HL^k^ index, 1-score increment | 0.13 (0.11 to 0.15) | <.001 | 1.04 (1.02-1.06) | <.001 | 1.02 (1-1.04) | .046 | 1.01 (0.99-1.03) | .179 | 1.03 (1.02-1.04) | <.001 | 0.97 (0.96-0.98) | <.001 |
| eHEAL^l^,1-score increment | 0.22 (0.18 to 0.25) | <.001 | 1.04 (1.02-1.07) | .002 | 1.03 (1-1.07) | .028 | 1.01 (0.98-1.04) | .531 | 1.04 (1.03-1.05) | <.001 | 0.96 (0.95-0.98) | <.001 |

^a^AIPC: adherence to infection prevention and control procedures.

^b^Analyzed using bivariate linear regression model, with reference group is “less healthy diet”, and test group is “unchanged or healthier diet”.

^c^Analyzed using bivariate logistic regression model, with reference group is “never, stopped, or less smoke”, and test group is “unchanged or more smoke”.

^d^Analyzed using bivariate logistic regression model, with reference group is “never, stopped, or less drink”, and test group is “unchanged or more drink”.

^e^Analyzed using bivariate logistic regression model, with reference group is “never, stopped, or less physical activity”, and test group is “unchanged or more physical activity”.

^f^S-COVID-19-S: suspected coronavirus disease-2019 symptoms including common symptom (fever, cough, dyspnea), less common symptom (myalgia, fatigue, sputum production, confusion, headache, sore throat, rhinorrhea, chest pain, hemoptysis, diarrhea, and nausea/vomiting).

^g^B: unstandardized regression coefficient.

^h^CI: confidence interval.

^i^OR: odds ratio.

^j^Frontline areas are outpatient department, emergency department, isolation areas, imaging and laboratory diagnosis department, patient administration areas.

^k^HL: health literacy.

^l^eHEAL: e-health literacy.
